# Characterization and functional validation of β-carotene hydroxylase *AcBCH* genes in *Actinidia chinensis*

**DOI:** 10.1093/hr/uhac063

**Published:** 2022-03-14

**Authors:** Hui Xia, Yuanjie Zhou, Zhiyi Lin, Yuqi Guo, Xinling Liu, Tong Wang, Jin Wang, Honghong Deng, Lijin Lin, Qunxian Deng, Xiulan Lv, Kunfu Xu, Dong Liang

**Affiliations:** 1 College of Horticulture, Sichuan Agricultural University, Chengdu 611130, China; 2 Institute of Pomology and Olericulture, Sichuan Agricultural University, Chengdu 611130, China

## Abstract

Carotenoids are the pigment substances of yellow-fleshed kiwifruit, and among them β-cryptoxanthin has only been detected in the brighter yellow-fleshed variety ‘Jinshi 1’. β-Carotene hydroxylase (BCH) catalyzes the formation of β-cryptoxanthin and zeaxanthin, but its molecular characteristics and functions have not been fully explained. Here we isolated two β-carotene hydroxylase genes, *AcBCH1* and *AcBCH2* from kiwifruit (*Actinidia chinensis*), and their relative expression levels exhibited a close correlation with the content of β-cryptoxanthin. *AcBCH1* catalyzed the formation of β-cryptoxanthin when transformed into β-carotene-accumulating yeast cells. Moreover, silenced expression of *AcBCH1* in kiwifruit caused decreases in the contents of zeaxanthin, lutein, and β-cryptoxanthin, and an increase in β-carotene content. The content of β-carotene decreased significantly after the *AcBCH1*/*2* genes were overexpressed in tomato. The content of zeaxanthin increased and β-carotene decreased in transgenic kiwifruit seedlings. The results will enrich our knowledge of the molecular mechanisms of carotenoid biosynthesis in kiwifruit.

## Introduction

Kiwifruit is a deciduous vine, which belongs to the genus *Actinidia*. Its fruit is rich in vitamins, minerals, dietary fiber, phenols, carotenoids, and other nutrients, thus enjoying the reputation of being the ‘king of fruits’ [1–4]. Initially, the green-fleshed cultivar ‘Hayward’ dominated the international kiwifruit market. With the successful breeding of yellow-fleshed cultivar ‘Hort16A’, its bright color and fresh taste immediately won the favor of consumers, and its market price is much higher than that of green-fleshed kiwifruit. The breeding of high-quality yellow-fleshed kiwifruit has become a new focus. Recently, Zespri International Ltd has launched a new yellow-fleshed cultivar, ‘SunGold’ (G3). In China, ‘Jinyan’, ‘Jintao’, ‘Jinshi 1’, and other yellow-fleshed varieties have also been bred. Carotenoids are the pigment substances of yellow-fleshed kiwifruit, and have a variety of biological functions. They endow the fruit with color, supply a source of vitamin A, remove free radicals generated by the body, enhance human immunity, and prevent some chronic diseases [5–9]. However, humans cannot synthesize carotenoids on their own; they only get them naturally through fruits and vegetables [10]. Therefore, the study of the carotenoid synthesis mechanism in fruits has become a hot issue.

The main biosynthetic pathways of carotenoids have been well elucidated in plants. Hygroscopic phosphoric acid [geranylgeranylpyrophosphate (GGPP)] produced by the methylerythritol phosphate (MEP) pathway provides an initial substrate for carotenoid biosynthesis [11], and is ultimately catalyzed to produce the red lycopene by a series of enzymes such as phytoene synthase (PSY), phytoene desaturase (PDS), ζ-carotene desaturase (ZDS), ζ-carotene isomerase (Z-ISO), and carotene isomerase (CRTISO) [12–15]. Cyclization of the lycopene chain terminus is divided into two branches. In the α-branch, an ε-ring and a β-ring are introduced at the ends of the carbon chain of lycopene to produce α-carotene under the action of lycopene ε-cyclase (LCYe) and lycopene β-cyclase (LCYb), respectively, [16]. Under the action of cytochrome P450 monooxygenase CYP97A or CYP97C, the hydroxylation of the β- and ε- rings of α-carotene produces lutein [17]. In the β-branch, LCYb catalyzes lycopene to introduce a β-ring at each end to form β-carotene, which is further transformed into β-cryptoxanthin and zeaxanthin under the action of β-carotene hydroxylase (BCH) [18]. Antheraxanthin and violaxanthin are produced by zeaxanthin epoxidase (ZEP) [19] catalysis. Zeaxanthin can be reconstituted by violaxanthin de-epoxidase (VDE) [20].

Lutein, β-carotene, and zeaxanthin are the main carotenoid components in kiwifruit; α-carotene appears only at the young fruit stage and decreases to an undetectable level as the fruit ripens [21]. In our previous studies, it was found that the flesh of ‘Jinshi 1’ was brighter and yellower than that of other yellow-fleshed varieties, such as ‘Jinyan’, at the ripening stage [22]. In addition to β-carotene, lutein, and zeaxanthin, β-cryptoxanthin was detected in the flesh of ‘Jinshi 1’ by HPLC, but was not detected in other varieties [22]. Therefore, we speculated that the accumulation of β-cryptoxanthin might be one of the main reasons for the yellower flesh color of ‘Jinshi 1’. β-Cryptoxanthin is catalyzed by β-BCH with β-carotenoid as substrate, and is further catalyzed by BCH to form zeaxanthin. In this study, two *AcBCH* genes were isolated from ‘Jinshi 1’ and overexpressed in tomato and kiwifruit to reveal its function in carotenoid biosynthesis. The results will supplement knowledge of the molecular mechanism of carotenoid metabolism in plants, and provide some reference for improving kiwifruit fruit quality by genetic engineering technology.

## Results

### Gene cloning and sequence analysis

**Figure 1:**
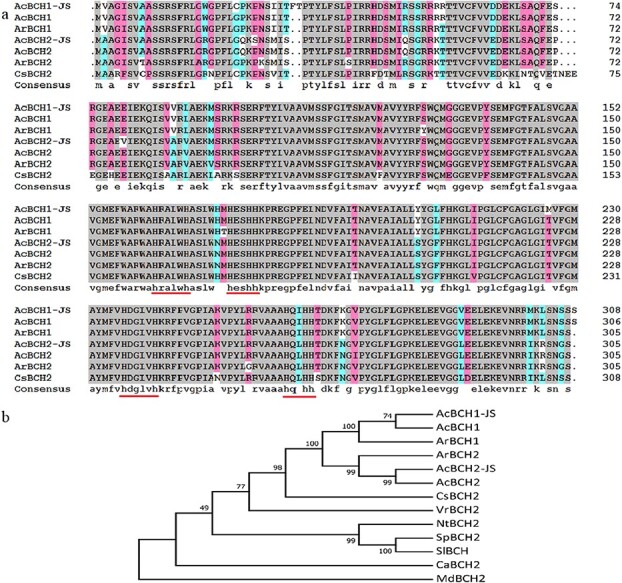
Sequence alignment (**a**) and phylogenetic tree (**b**) of BCHs from different plants. AcBCH1-JS, ‘Jinshi 1’; AcBCH2-JS, ‘Jinshi 1’; AcBCH1, *Actinidia chinensis* var. *chinensis*, PSS33035.1; ArBCH1, *Actinidia rufa*, GFY84155.1; AcBCH2, *A. chinensis* var. chinensis, PSS04390.1; ArBCH2, *A. rufa*, GFZ06448.1; CsBCH2, *Camellia sinensis*, XP_028112187.1; VrBCH2, *Vitis riparia*, XP_034710058.1; NtBCH2, *Nicotiana tabacum*, NP_001312763.1; SpBCH2, *Solanum pennellii*, XP_015080043.1; SlBCH, *Solanum lycopersicum*, NP_001234348.1; CaBCH2, *Coffea arabica*, XP_027110221.1; MdBCH2, *Malus domestica*, XP_008343769.2.

Two BCH genes were successfully isolated from ‘Jinshi 1’ and named as *AcBCH1* and *AcBCH2*. The full-length open reading frames of *AcBCH1* and *AcBCH2* were 927 and 918 bp, encoding 308 and 305 amino acids, respectively. Sequence alignment revealed that AcBCH1 from ‘Jinshi 1’ has a phenylalanine (F) and a threonine (T) inserted at amino acid position 31 compared with other BCH amino acid sequences, and AcBCH1 (PSS33035.1) has an extra serine (S) at the C-terminus. The intermediate and terminal sequences of AcBCH1 and AcBCH2 were highly conserved and contained the conserved domains ‘HXXXXH’ and ‘HXXHH’ (underlined in red in Fig. 1a), which were related to carotenoid hydroxylase activity. The phylogenetic analysis of BCH among different species showed that AcBCH1 and AcBCH2 were clustered on a single branch with the BCH of *Actinidia*, and were most closely related to CsBCH2 of *Camellia sinensis* (Fig. 1b), which was consistent with their genetic relationship.

### Expression pattern of *AcBCH1*/*2* in kiwifruit

**Figure 2:**
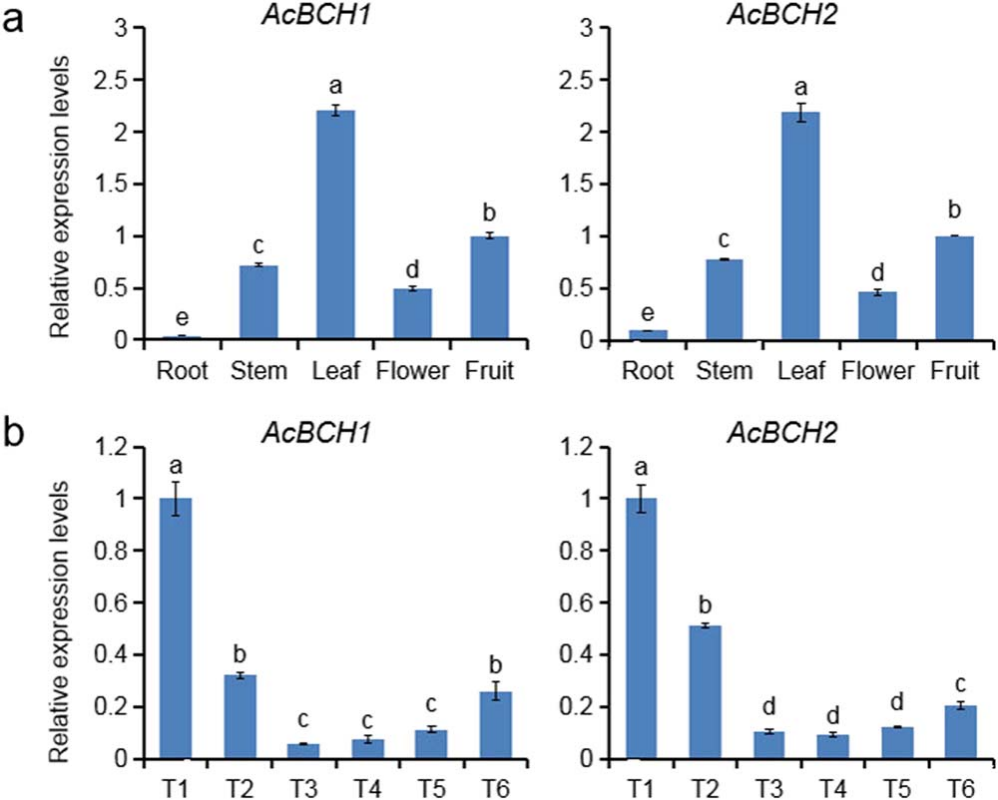
Spatiotemporal expression pattern of *AcBCH1*/*2*. **a** Relative expression levels of *AcBCH1*/*2* in different tissues of kiwifruit. **b** Relative expression levels of *AcBCH1*/*2* in kiwifruit during fruit development. T1–T6 represent 30, 55, 70, 95, 130, and 145 days after 75% flower drop. Each value is expressed as mean ± standard deviation (*n* = 3). Different lower-case letters indicate significant differences at *P* < .05 level by Studentʼs *t*-test.

qRT–PCR analysis showed that *AcBCH1* and *AcBCH2* were expressed in all collected tissues, with high abundance of expression in leaves and fruits, followed by stems and flowers, and almost no expression in roots (Fig. 2a). During fruit development, the expression levels of *AcBCH1* and *AcBCH2* continued to decrease in the early stages (T1–T3), but gradually increased after the color-turning stage (Fig. 2b), when β-cryptoxanthin also began to accumulate in the fruit (Supplementary Data Fig. S1). Among the 12 genes involved in other carotenoid metabolic pathways, only *LCYb2*, *ZEP*, *CCD* and *NCED* showed an upward trend in expression after color turning (Supplementary Data Table S2).

Pearson correlation analysis revealed that the relative expression levels of *LCYb2*, *ZEP*, *CCD*, and *NCED* were positively correlated with β-cryptoxanthin content (Supplementary Data Table S2), but none of these genes were structural genes directly involved in β-cryptoxanthin synthesis. The relative expression levels of *AcBCH1* and *AcBCH2* were highly significantly positively correlated with β-cryptoxanthin content (*r* = .824 and .875, respectively) and negatively correlated with β-carotene content (*r* = −.618 and −.688, respectively) during late fruit development, which suggested that *AcBCH1* and *AcBCH2* were closely related to the synthesis of β-cryptoxanthin in ‘Jinshi 1’ fruit.

### Enzymatic activity analysis of AcBCHs in yeast

**Figure 3:**
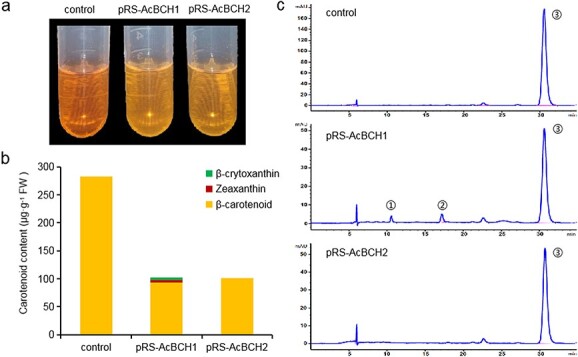
Changes of carotenoid content in yeast. **a** Color change of carotenoid extracts from yeast. **b** Carotenoid content and components of yeast. **c** HPLC analysis of carotenoid accumulation in yeast: (1) zeaxanthin; (2) β-cryptoxanthin; (3) β-carotene.

When *AcBCH1* and *AcBCH2* were expressed in β-carotenoid-only-producing yeast, the extracts from yeast containing pRS-AcBCH1/2 were significantly more yellow than untreated yeast, which appeared orange (Fig. 3a). Determination of yeast carotenoid content by HPLC revealed that yeast transformed with *AcBCH1* contained small amounts of β-cryptoxanthin and zeaxanthin in addition to β-carotene, indicating that AcBCH1 has β-carotene hydroxylase activity (Fig. 3b and c). In yeast extracts transferred with *AcBCH2*, the content of β-carotene was significantly reduced compared with the control, as was the case with yeast strains transferred with *AcBCH1*, but no β-cryptoxanthin and zeaxanthin were detected. We speculated that the enzyme activity of AcBCH2 was weaker than that of AcBCH1, and the downstream product content was too low to be detected. Only small amounts of β-cryptoxanthin and zeaxanthin were detected in AcBCH1-overexpressing yeast (Fig. 3b and c).

### Function validation of *AcBCH1*/*2* in kiwifruit based on virus-induced gene silencing

**Figure 4:**
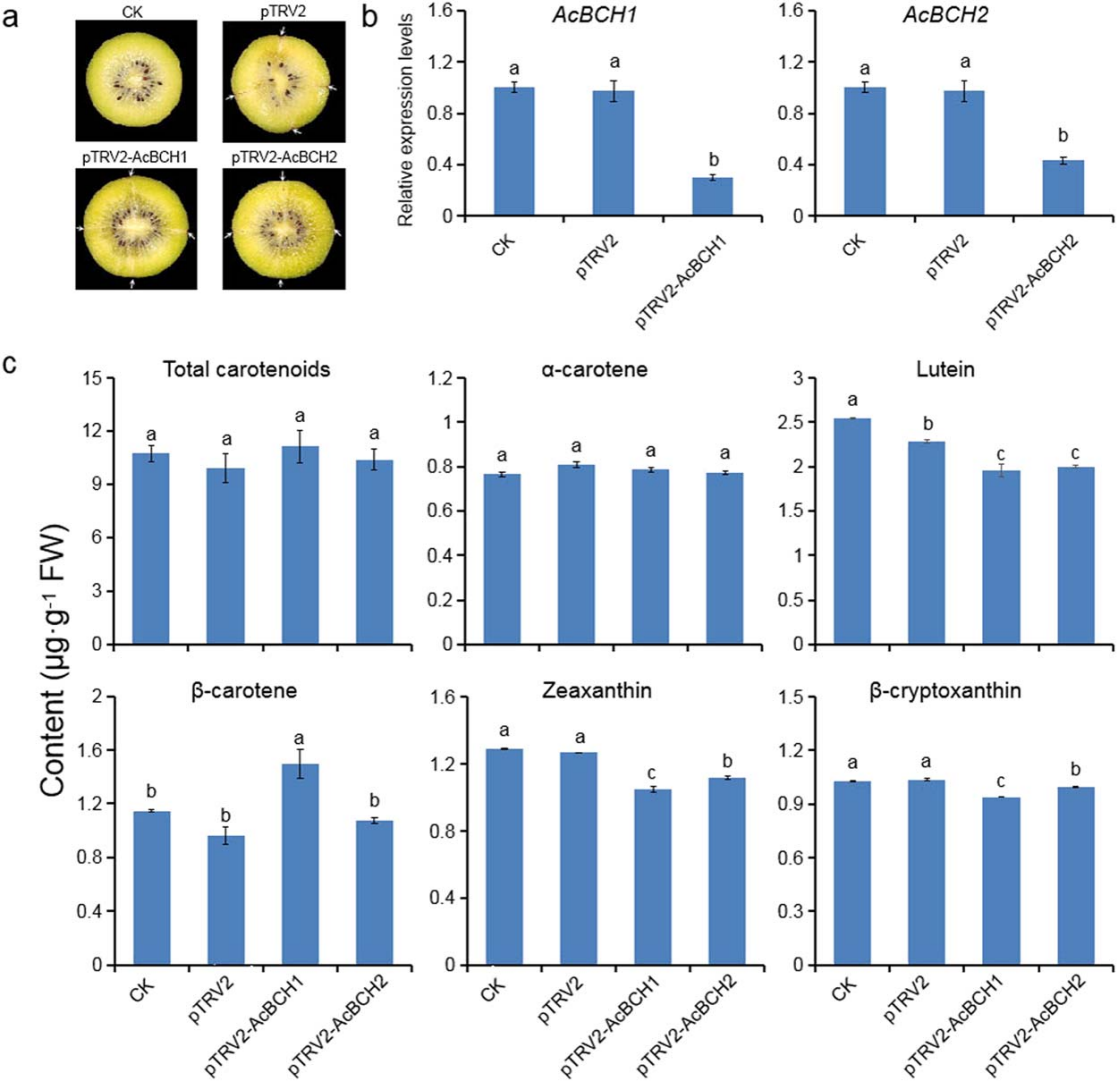
Virus-induced *AcBCH1*/*2* silencing in fruit of ‘Jinshi 1’. **a** External appearance of TRV-inoculated kiwifruit. White arrows indicate injection sites. **b** Relative expression of *BCH* gene. **c** Carotenoid content. Each value is expressed as mean ± standard deviation (*n* = 3). CK, untreated control. Different lower-case letters indicate significant differences at *P* < .05 level by Studentʼs *t*-test.

Virus-induced gene silencing (VIGS) was applied to suppress *AcBCH1* and *AcBCH2* expression in ‘Jinshi 1’ fruit at the time of color break. The expression of *AcBCH1* and *AcBCH2* 1 week after infiltration of fruit with pTRV2-AcBCH1 and pTRV2-BCH2 was significantly suppressed, although there was no significant difference in visual appearance (Fig. 4a and b). The carotenoid content of the fruit was further measured. Compared with untreated and negative control infiltrated with pTRV2, the contents of lutein, β-cryptoxanthin, and zeaxanthin in both pTRV2-ACBCH1- and pTRV2-ACBCH2-treated fruits were significantly reduced, but the contents of total carotenoids and α-carotene were not significantly different (Fig. 4c). The content of β-carotene in pTRV2-ACBCH1-treated fruits was significantly increased, while it was not increased in pTRV2-ACBCH2 treated fruits compared with control (Fig. 4c), which was consistent with our previous hypothesis that AcBCH2 was less active than AcBCH1. These results indicated that silencing expression of *AcBCH1* and *AcBCH2* prevented the conversion of β-carotene to β-cryptoxanthin and zeaxanthin, leading to the accumulation of β-carotene, and had the potential to affect carotenoid metabolism in the α-branch, thus reducing lutein synthesis.

### *AcBCH1*/*2* overexpression in tomato

**Figure 5:**
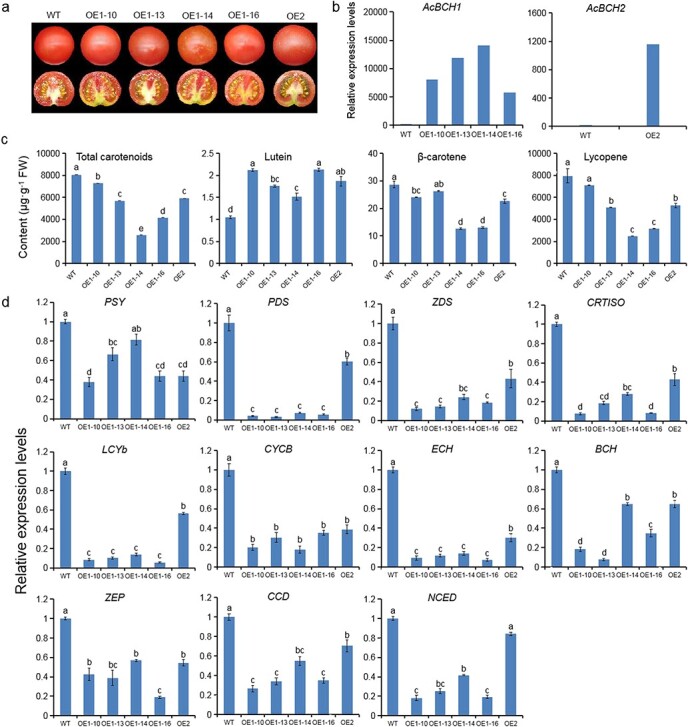
Overexpression *AcBCH1*/*2* in tomato. **a** Phenotype of transgenic tomato. **b** Relative expression levels of *AcBCH1/2*. **c** Carotenoid content in transgenic tomato. **d** Relative expression of carotenoid biosynthetic genes. Each value is expressed as mean ± standard deviation (*n* = 3). Different lower-case letters indicate significant differences at *P* < .05 level by Studentʼs *t*-test.

To further examine the function of *AcBCH1/2* in carotenoid biosynthesis of fruit, constructs of pBI121-AcBCH1/2 were transformed into tomato, which is a good fleshy fruit model plant. A total of four independent T2-generation transgenic plants (OE1-10, OE1-13, OE1-14, and OE1-16) with high levels of *AcBCH1* transcripts and one independent T2-generation transgenic plant (OE2) with high levels of *AcBCH2* transcripts were obtained. Compared with the wild-type tomatoes, the transgenic tomatoes showed no significant color changes at ripening, except for the yellowish color of OE1-14 (Fig. 5a). The main carotenoids accumulating in ripening tomato fruit were lycopene, β-carotene, and lutein. *AcBCH1*/*2*-overexpressing tomatoes had significantly lower lycopene and β-carotene contents and significantly higher lutein content than wild-type tomatoes (Fig. 5c). We performed qRT–PCR analysis of the main structural genes of the endogenous carotenoid metabolic pathway in transgenic tomato fruits. The relative expression levels of the majority of both upstream (*PSY*, *PDS*, *ZDS*, *CRTISO*, *LCYb*, *CYCB*) and downstream (*ZEP*, *CCD*, *NCED*) genes of *BCH* were reduced in *AcBCH1*/*2*-overexpressing tomatoes fruits at maturity (Fig. 5d). These results suggested that overexpression of *AcBCH1* and *AcBCH2* produced a large amount of β-carotene hydroxylase, leading to the consumption of β-carotene and lycopene and the production of lutein. Meanwhile, overexpression of heterologous *AcBCH* genes may contribute to the differential accumulation of carotenoid metabolites and consequently to changes in the transcript levels of a large number of endogenous carotenoid biosynthetic genes.

### *AcBCH1*/*2* overexpression in ‘Hongyang’ kiwifruit

**Figure 6:**
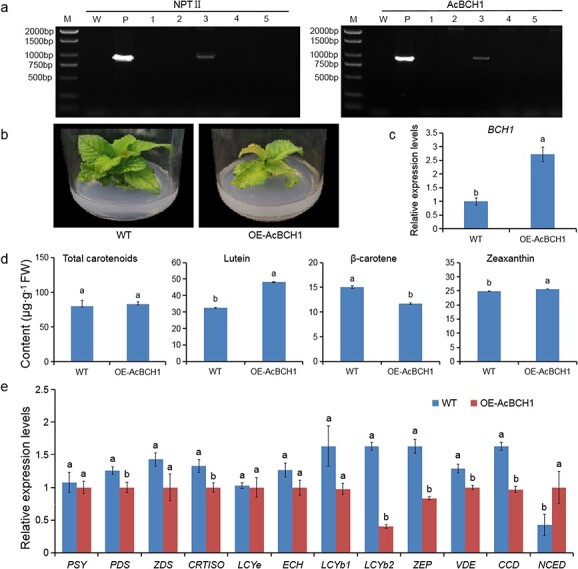
Overexpression *AcBCH1*/*2* in ‘Hongyang’ kiwifruit. **a** PCR amplification of *NPT II* and *AcBCH1*. **b** Phenotype of transgenic kiwifruit. **c** Relative expression of *BCH1*. **d** Carotenoid content. **e** Relative expression of carotenoid biosynthetic genes. Each value is expressed as mean ± standard deviation (*n* = 3). Different lower-case letters indicate significant differences at *P* < .05 level by Studentʼs *t*-test.

To further confirm whether *AcBCH1*/*2* could modulate carotenoid accumulation, pBI121-AcBCH1 and pBI121-AcBCH2 were transformed into ‘Hongyang’ kiwifruit. PCR amplification of the resistance gene *NPT I* and the target genes *AcBCH1*/*2* was carried out using the resistant bud genomic DNA as template, the pBI121-AcBCH1/2 plasmid as a positive control, and the non-transgenic material as a negative control. As shown in Fig. 6a, only one independent line overexpressing *AcBCH1* was obtained. The relative expression levels of *AcBCH1* in the transgenic plants were significantly higher than those of the wild type (Fig. 6c), further demonstrating that transgenic plant overexpressing *AcBCH1* were obtained. Compared with wild-type plants, OE-AcBCH1 plants had weaker growth and yellowish leaves (Fig. 6b). Carotenoids in the leaves of the transgenic plants were measured in a preliminary investigation, considering the long nutritional growth cycle of kiwifruit and the unavailability of fruit in the short term. Lutein, β-carotene, and zeaxanthin were identified as the main carotenoids in transgenic plant leaves using HPLC (Fig. 6d). Lutein and zeaxanthin concentrations were significantly increased and β-carotene content was significantly reduced in transgenic plant leaves, but total carotenoid content was not significantly changed, compared with non-transgenic plants (Fig. 6d). Most genes of carotenoid metabolism in transgenic kiwifruit leaves were downregulated in expression, whereas *NCED* was significantly upregulated in expression (Fig. 6e).

## Discussion

Carotenoids are essential secondary metabolites in plants and have an important influence on the color diversity of horticultural plants. β-Cryptoxanthin contributes significantly to the yellow pigmentation of horticultural plants. For example, β-cryptoxanthin pigmentation levels were much higher in yellow-fleshed varieties of raspberry and papaya than in red-fleshed varieties [28, 29]. Our previous research found that β-cryptoxanthin may be responsible for the brighter yellow flesh of ‘Jinshi 1’ [30]. In this study, we also found that the carotenoid extracts of yeast that had accumulated β-cryptoxanthin became more yellowish in color than the extracts of yeast that originally appeared orange.

The accumulation of carotenoids is regulated by numerous factors, of which structural genes are among the most critical. The expression trend of *CpCHX-β* and *CiHYb* (*BCH* homologous gene in *Carica papaya* and *Citrus* spp. cv. ‘Seinannohikari’) during fruit ripening was found to be consistent with the increasing trend of β-cryptoxanthin accumulation, and the high expression levels of *CpCHX-β* and *CiHYb* were thought to be responsible for the accumulation of β-cryptoxanthin in both papaya and citrus [31, 32]. Similar results were observed in our study: *AcBCH1* and *AcBCH2* expression were significantly positively correlated with β-cryptoxanthin content, while negatively correlated with β-carotene content (Supplementary Data Table S2). The β-carotene hydroxylase gene of *Arabidopsis* was abundantly expressed in all photosynthetic tissues [33]; in tomato, *CrtR-b1* (*BCH1* homologous gene) showed a constitutive expression pattern [34]. In this study, similarly *AcBCH1* and *AcBCH2* were constitutively expressed genes with particularly high abundance in leaves and fruits (Fig. 2a). These findings implied the possibility that *AcBCH1* and *AcBCH2* were important genes for β-cryptoxanthin synthesis in kiwifruit. Thus, we performed a series of experiments to characterize and validate the function of *AcBCH1*/*2* in carotenoid metabolism.

Multiple amino acid sequence alignment revealed that AcBCH1 and AcBCH2 contained four typical histidine structural domains (Fig. 1a), the absence or alteration of which would result in the disruption of enzyme activity [35]. We further investigated the enzymatic activity of *AcBCH1* and *AcBCH2* using β-carotene-accumulating yeast. When *AcBCH1* was expressed in yeast, β-carotene was converted to small amounts of β-cryptoxanthin and zeaxanthin, which supported the result that *AcBCH1* was a key gene that catalyzes β-carotene conversion in kiwifruit fruit (Fig. 3). CrCYP97A, which is involved in the hydroxylation of α-carotene in sweet orange, was reported to have no enzymatic activity in an enzyme activity test in α-carotene-accumulating *Escherichia coli*, because the carotenoid components and concentration did not change compared with control [36]. However, in this study, when *AcBCH2* was overexpressed in yeast, no accumulation of hydroxycarotene was detected, but β-carotene was significantly reduced (Fig. 3). Thus it was speculated that *AcBCH2* had β-carotene hydroxylase activity, but it was weaker than that of *AcBCH1*.

The VIGS system has been successfully applied in many horticultural plants, such as loquat [37], apple [38], and kiwifruit [25], and has successfully contributed to the silencing of target genes. In California poppy, when the *BCH* was silenced with the VIGS system the conversion of β-carotene was significantly blocked and downstream β,β-xanthophylls such as zeaxanthin almost disappeared [39]. In the present study, we successfully inhibited endogenous *BCH* expression in ‘Jinshi 1’ kiwifruit fruit using the VIGS system. The transcript levels of *AcBCH1* and *AcBCH2* were significantly downregulated, resulting in β-carotene accumulation and a significant reduction in β-cryptoxanthin and zeaxanthin (Fig. 4). These results indicated that *AcBCH1* and *AcBCH2* were key genes participated in the step of β-carotene hydroxylation to produce β-cryptoxanthin and zeaxanthin.

*CrtR-b2* (*BCH2*) has been reported to be overexpressed in tomato, resulting in significantly lower lycopene, β-carotene, and total carotenoid production than wild-type [40]. Similarly, in the present study overexpression of *AcBCH1* and *AcBCH2* in ripe tomatoes resulted in decreases in β-carotene, lycopene, and total carotenoid concentrations (Fig. 5c). In potato with *CHY1* and *CHY2* silenced by RNAi technology, most of the carotenogenic genes were upregulated [41]. In carrot *AtCYP97A*-overexpressing lines, blocked translation levels of *PSY* resulted in reduced total carotenoid content [42]. However, in this study, most carotenoid biosynthetic genes (*PSY*, *PDS*, *ZDS*, *CRTISO*, *LCYb*, *CYCB*, *ECH*, *BCH*, *ZEP*, *CCD*, and *NCED*) were negatively regulated in the presence of overexpression of *AcBCH1* and *AcBCH2* in transgenic tomatoes (Fig. 5d). In particular, downregulation of *PSY*, the primary rate-limiting enzyme gene in the plant carotenoid pathway [43], explained the reduction in total carotenoids in tomatoes overexpressing *AcBCH1* and *AcBCH2*. The reduction in the relative expression level of endogenous *BCH* may be due to the competitive effect of the heterologous gene on the substrate.

In this study, we fortunately obtained a transgenic kiwifruit line overexpressing *AcBCH1*. As kiwifruit is a perennial woody tree with a long juvenile period, it is difficult to obtain fruit in a short period of time. Therefore, the role of AcBCH1 in carotenoid synthesis was preliminarily investigated by measuring the carotenoid content in the leaves of transgenic plants. Overexpression of *AcBCH1* in kiwifruit leaves promoted the conversion of β-carotene to hydroxylated derivatives, lutein and zeaxanthin (Fig. 6d). This was consistent with the recently reported reduction in β-carotene after overexpressing carrot *DcBCH1* in *Arabidopsis* [44]. There was no significant change in the amount of total carotenoid in transgenic kiwifruit leaves compared with the wild type, which may be attributed to the insignificant change in the transcription level of the *PSY* gene, as PSY has been reported to be closely related to the total amount of carotenoid flow [5, 43]. The transcript levels of *NCED* were significantly elevated in transgenic kiwifruit (Fig. 6e), suggesting that overexpression of *AcBCH1* may promote abscisic acid synthesis. Based on the above findings, we determined that *AcBCH1* and *AcBCH2* were the key genes of β-carotene hydroxylation and regulated carotenoid metabolism to a certain extent.

*BCH1*/*2* is the main structural gene responsible for β-carotene hydroxylation, while *CYP97A* and *CYP97C* are key genes controlling the hydroxylation of α-carotene to produce ε,β-xanthophylls such as lutein in plants. However, in an *Arabidopsis* mutant lacking *CYP97A*, lutein synthesis was not completely suppressed [45]. In *Arabidopsis* mutants lacking *CHY1* and *CHY2* (*BCH1* and *BHC2*), lutein no longer accumulated [46]. In *Citrus Csβ-CHX* (*BCH*) interference lines, ε,β-xanthophyll accumulation was drastically reduced [47]. All these observations suggested that the substrates of hydroxylase are not necessarily highly specific. In this study, we discovered that lutein content was significantly reduced in *AcBCH1*/*2*-silenced ‘Jinshi 1’ kiwifruit fruit, and markedly increased in *AcBCH1*/*2*-overexpressing tomato fruit or ‘Jinshi 1’ kiwifruit leaves (Figs 4c, 5c, and 6d). These results pointed to the possibility that *AcBCH1* and *AcBCH2* may assume a partial role in the hydroxylation of α-carotene.

In conclusion, we identified two β-carotene hydroxylase genes in ‘Jinshi 1’ kiwifruit, named *AcBCH1* and *AcBCH2*. *AcBCH1* and *AcBCH2* are mainly involved in the hydroxylation of β-carotene, and may also have potential regulatory roles in the metabolism of hydroxylated derivatives downstream of α-carotene. Overexpression of *AcBCH1* and *AcBCH2* in tomato reduces lycopene, β-carotene, and total carotenoid contents. Overexpressing *AcBCH1* in kiwifruit reduces β-carotene content and increases hydroxylation products. This research is valuable for molecular breeding to improve kiwifruit quality. In the future, we will attempt to knock down *AcBCH1* and *AcBCH2* using CRISPR/Cas9 to investigate their functions in greater depth in kiwifruit fruit. Furthermore, we will combine sequence analyses of promoters to search for upstream transcription factors targeting *AcBCH1* and *AcBCH2*, with a view to expanding the understanding of the regulatory network of carotenoid metabolism.

## Materials and methods

### Plant materials

Samples of leaves, roots, stems, flowers, and fruit of ‘Jinshi 1’ (*Actinidia chinensis*) were collected from the kiwifruit research base of the Sichuan Academy of Natural Resources Science, Jiandi Town, Shifang City, Sichuan Province (104°2′E, 31°23′N). Fruits were collected at 30, 55, 70, 95, 130, and 145 days after 75% flower drop. All samples were quick-frozen in liquid nitrogen and stored at −80°C.

Sterile kiwifruit seedlings (*A. chinensis* cv. ‘Hongyang’) were maintained in our laboratory and subcultured on Murashige and Skoog (MS) medium supplemented with 1.0 mg l^−1^ 6-benzylaminopurine (6-BA) and 0.1 mg l^−1^ naphthaleneacetic acid (NAA). Tomato (*Solanum lycopersicum* cv. ‘Alisa Craig’) seeds were kindly given by Dr Dawei Li (Northwest A & F University).

### RNA extraction and qRT–PCR analysis

The total RNA of all samples was extracted using the Plant RNA Extraction Kit-V1.5 (Biofit, Chengdu, China). Then the cDNA was synthesized using the PrimeScript™ RT reagent Kit with gDNA Eraser (Perfect Real Time) kit (TaKaRa, Dalian, China). The qRT–PCR primers for genes participating in carotenoid biosynthesis in kiwifruit were designed based on the sequences acquired from the kiwifruit genome database (http://kiwifruitgenome.org/). The specific primers used for qRT–PCR for tomato were as previously reported [23]. The qRT–PCR reaction was performed using the CFX96 Real-Time System C1000 Thermal Cycler (Bio-RAD, Hercules, CA, USA) under the recommended conditions provided by TB Green Premix Ex Taq II (Takara, Dalian, China). The relative expressions of the target genes were calculated using the 2^-ΔΔCt^ method with *Actin* (EF063572) as the reference gene. The primer sequences are listed in Supplementary Data Table S1.

### Gene cloning and sequence analysis

The complete coding sequences of *AcBCH* genes were cloned from ‘Jinshi 1’ fruits using specific primers (Supplementary Data Table S1) based on sequences obtained from the kiwifruit genome database. A series of BCH protein sequences from other species was derived from GenBank. DNAMAN and MEGA7.0 were used for multiple sequence alignment and phylogenetic tree construction, respectively.

### Transformation of yeast engineering bacteria

The yeast expression vector pRS423 and the β-carotene-accumulating yeast strain (*Saccharomyces cerevisiae*) were provided by Dr Zhiyong Yue (Xi’an Foreign Affairs University). New *AcBCH1* and *AcBCH2* nucleic acid sequences were optimized and synthesized based on the yeast codon preference. The pRS423 vector fragment and the optimized target gene were cloned separately using specific primers (Supplementary Data Table S1) with homologous recombinant sequences, then mixed at a molar concentration of 1:1 and transformed into yeast using the method described by Yamada *et al*. [24]. After incubation on CM-His solid medium at 30°C for 3 days, DNA from the yeast was extracted using the Yeast Genomic DNA Extraction Kit (TIANGEN, China) for PCR to determine whether positive colonies were obtained. The yeast containing pRS423-AcBCH1/2 was picked and incubated in CM-His liquid medium at 30°C with shaking until OD600 reached 1, after which the organisms were collected by centrifugation.

### Virus-induced gene silencing in kiwifruit

The vectors pTRV1 and pTRV2, based on the modified tobacco rattle virus, were kindly donated by Dr Qing Chen (Sichuan Agricultural University). The 400–600 bp conserved fragments of *AcBCH1* and *AcBCH2*, respectively, were cloned and inserted into the pTRV2 vector. The pTRV2-AcBCH1, pTRV2-AcBCH2, and empty vectors pTRV1 and pTRV2 were then transformed into *Agrobacterium tumefaciens* strain GV3101, respectively, using the freeze–thaw method and incubated in LB liquid medium at 28°C. After 24 h, the *Agrobacterium* cells were harvested, and then resuspended in buffer (10 mmol l^−1^ MgCl_2,_ 10 mmol l^−1^ 2-(N-Morpholinyl)ethanesulfonic acid (MES), and 200 μmol l^−1^ acetosyringone (AS), pH 5.6), adjusting the OD600 to 1. The suspensions of *Agrobacterium* containing pTRV2-AcBCH1, pTRV2-AcBCH2, and pTRV2 were mixed with *Agrobacterium* containing pTRV1 in a ratio of 1:1, respectively, and held for 2–3 h prior to injection.

Sixty fruits of ‘Jinshi 1’ were picked at the color-turning stage (flesh color was pale green), and divided into four groups for injection treatment. The mixture was injected in four directions perpendicular to the longitudinal diameter from the middle of the fruit using the method of Liu *et al*. [25]. Four locations at the equator of each fruit were selected for injection of 400 μl of the mixture slowly in the central direction to a depth of ~2 cm. The injection was repeated every other day for a total of three times at the same location, and placed in a constant-temperature incubator at 25°C. On the 7th day after treatment, rotted fruits were removed, and the remaining fruits were cut open, and the pulp within 1 cm around the injection point was sampled. Samples from at least three fruits were mixed together as a replication, with three replications.

### Construction of overexpression vector

The coding regions of *AcBCH1* and *AcBCH2* were amplified with the special primers listed in Supplementary Data Table S1. The amplification products were ligated into pBI121 vector to form two CaMV35S promoter-driven overexpression vectors, pBI121-AcBCH1 and pBI121-AcBCH2. These vectors were transferred into *Agrobacterium* GV3101 using the freeze–thaw method.

### Transformation of tomato

The constructs pBI121-AcBCH2 and pBI121-AcBCH1 were transformed into tomatoes according to the method described by Sun *et al*. [26] with slight modification. Freshly expanded tomato cotyledons with both ends excised were suspended in *Agrobacterium* suspension for 30 minutes, then inoculated on MS solid medium supplemented with 1.5 mg l^−1^ zeaxanthin and 0.5 mg l^−1^ IAA (indoleacetic acid) with the lower leaf surface downwards. After 2 days of incubation at 28°C protected from light, the leaves were transferred to MS medium containing 1.5 mg l^−1^ zeaxanthin, 0.5 mg l^−1^ IAA, 300 mg l^−1^ Timentin, and 100 mg l^−1^ kanamycin. When the adventitious shoots reached 3–4 cm, they were inoculated onto MS medium supplemented with 0.2 mg l^−1^ IAA, 300 mg l^−1^ Timentin, and 100 mg l^−1^ kanamycin to induce rooting. Intact plants were obtained and transplanted into nutrient bowls after 1–2 weeks and placed in a greenhouse for normal management. Six fruits from each T2 generation individual plant were collected for the determination of relevant indicators, and groups of two fruits were mixed as a replicate for three biological replicates.

### Transformation of ‘Hongyang’ kiwifruit

The transformation of ‘Hongyang’ kiwifruit was performed as described by Wang *et al*. [27]. The leaves or petioles were precultured for 3 days on MS medium containing 1.0 mg l^−1^ 6-BA and 0.1 mg l^−1^ NAA, then transferred to an *Agrobacterium* suspension by immersion for 10 min. The precultured materials were cocultivated with *Agrobacterium* for 2 days in dark on MS medium consisting 100 μmol l^−1^ AS，1.0 mg l^−1^ 6-BA and 0.1 mg l^−1^ NAA. Afterwards, the explants were inoculated into MS medium supplemented with 1.0 mg l^−1^ 6-BA, 0.1 mg l^−1^ NAA, 20 mg l^−1^ kanamycin, and 400 mg l^−1^ carbenicillin to induce callus information. After 20 days, the calluses obtained were transferred to a histoculture chamber with a light intensity of 90 μmol m^−2^ s^−1^, a photoperiod of 16 h light/8 h dark, a temperature of 25 ± 2°C, and a relative humidity of 75% for normal light culture.

### Carotenoid extraction and measurement

The extraction and determination of carotenoids was carried out as previously described [22]. One gram of powdered sample was added to 5 ml of acetone extract solution containing 0.1% butylated hydroxytoluene and sonicated for 60 min. After centrifugation at 4°C, the supernatant was filtered through a 0.22 μm filter for further determination. Total carotenoids were determined using a spectrophotometer (UV-1200, Mapada, Shanghai, China) at 450 nm. The carotenoid components were determined using an HPLC system (Agilent 1260, Agilent Technologies, Palo Alto, CA, USA) equipped with a VWD (variable wavelength scanning UV detector) and a YMC C30 column (250 mm × 4.6 mm, 5 μm). The mobile phase was methyl tertbutyl ether:methanol (30:70 V/V) at a flow rate of 0.5 ml/minute at a column temperature of 25°C. The injection volume and detection wavelength were set as 20 μl and 450 nm, respectively. The standard curves of six standard samples (α-carotene, lycopene, lutein, β-carotene, β-cryptoxanthin, and zeaxanthin) were established by an external standard method. The retention time and peak area of standard samples were used to qualitatively and quantitatively determine carotenoid components.

### Statistical analysis

All data are expressed as mean ± standard error of three replicates. Statistical significances of differences were determined and Pearson correlation analysis was performed using SPSS (version 20.0, SPSS Inc., Chicago, IL, USA).

## Acknowledgements

This work was supported by the Sichuan Science and Technology Department Project (2021YFYZ0010).

## Author contributions

D.L. and H.X. conceived the experiments. Y.Z., Z.L., Y.G., X.L., T.W., J.W., and H.D. carried out the experiments with the help of L.L. and K.X. Q.D. and X.L. contributed the plant materials and data analysis. Y.Z. and H.X. wrote the manuscript and D.L. edited the manuscript.

## Data availability

All relevant data are included in the paper and its supplementary files.

## Conflict of interest

The authors declare no competing interests.

## Supplementary data

Supplementary data is available at *Horticulture Research* online.

## Supplementary Material

Web_Material_uhac063
