# Supplementary material for: Characterization and functional validation of β-carotene hydroxylase AcBCH genes in Actinidia chinensis
Source: Hortic Res. 2022 Mar 14;9:uhac063. doi: 10.1093/hr/uhac063 (PMC9123235; doi:10.1093/hr/uhac063)
Supplement: Web_Material_uhac063 [file web_material_uhac063.docx]

**Supplementary Table [S1](https://www.nature.com/articles/s41438-020-00379-w#MOESM1). The primers used in the study.**

| Name | Primer sequences (5’→3’) | Notes |
| --- | --- | --- |
| AcBCH1-F | CTCTAAGCTGCCCAAACTCC | Gene cloning |
| AcBCH1-R | CGTAGCCAAGCGAGAATTTTA |  |
| AcBCH2-F | CTACTAAAACCACGCCAATCC |  |
| AcBCH2-R | CCCGTTAATTAGGCATTGCT |  |
| Action-qPCR-F | GCAGGAATCCATGAGACTACC | qRT-PCR primers of carotenoid synthesis genes in kiwifruit |
| Action-qPCR-R | GTCTGCGATACCAGGGAACAT |  |
| BCH1-qPCR-F | ACGAGTCTCATCATAAACCCAG |  |
| BCH1-qPCR-R | TGTGGAATAAACCGTAGTAGAGG |  |
| BCH2-qPCR-F | ACGAGTCTCATCATAAACCCAG |  |
| BCH2-qPCR-R | ATCCCAAACACCGTTATTCC |  |
| PSY-qPCR-F | TATAACGCTGCCTTGGCTCT |  |
| PSY-qPCR-R | TTTCCGGCAAATATGTCCTC |  |
| PDS-qPCR-F | CTTTGCATGCCAATAGCAGA |  |
| PDS-qPCR-R | GTCGGACTTCACCACCAAGT |  |
| ZDS-qPCR-F | TGTCCAAAGGTGGTACACGA |  |
| ZDS-qPCR-R | AGCAACAGGATCCCACATTC |  |
| CRTISO-qPCR-F | AGGTTTTGCCACCAGATACG |  |
| CRTISO-qPCR-R | ACCATGTGGCTTCTCCAAAC |  |
| LCYb1-qPCR-F | AATAGATCGGAGACGCCAGAG |  |
| LCYb1-qPCR-R | CCATGCCAATAACGAGGTTC |  |
| LCYb2-qPCR-F | CCCTCGCCACTTTCTATGT |  |
| LCYb2-qPCR-R | TTTCGGCTTACCCTACCAT |  |
| LCYe-qPCR-F | CTCAATGCAAGCTTGGAACA |  |
| LCYe-qPCR-R | CCAACTGGGTAAACGGAAGA |  |
| ECH-qPCR-F | TAGCAAGCAACTCCGTGATG |  |
| ECH-qPCR-R | ACTGCAGCTGATGTTTCGTG |  |
| ZEP-qPCR-F | CCCAGGAAGCCTCATATTCA |  |
| ZEP-qPCR-R | TTACCAGCGGGACCATCTAC |  |
| VDE-qPCR-F | TTTTGCCTCTGAGCATTGTG |  |
| VDE-qPCR-R | TCTACAAGGGGTGGTTCAGG |  |
| CCD-qPCR-F | AATGGAACTGGGAACACAGC |  |
| CCD-qPCR-R | TGAGCAGTGAAGGAATGTGC |  |
| NCED-qPCR-F | CACTTGCAAAACCCACAATG |  |
| NCED-qPCR-R | ATTCGACCCACTTGATCTCG |  |
| BCH1-yeast-F | AAACTATATCAATTAATTTGAATTAACaagcttAGGT  TCAAGAAGATGAGTTGGACAGC | Enzyme activity test |
| BCH1-yeast-R | ataccaagcatacaatcaactatctcatatacaATTCATGGTTGCCG  GTATTTCTGTTG |  |
| BCH2-yeast-F | ataccaagcatacaatcaactatctcatatacaATTCATGGCTGCTG  GTATTTCTGTTG |  |
| BCH2-yeast-R | AAACTATATCAATTAATTTGAATTAACaagcttAGGT  TCAAGAACCGTTAGACCTCTTG |  |
| pRS-BCH1-F | AAGGATGAAGCTGTCCAACTCATCTTCTTG  AACCTaagcttGTTAATTCAAATTAATTG |  |
| pRS-BCH1-R | AGAAGCAGCAACAGAAATACCGGCAACCA  TGAATtgtatatgagatagttgattgtatg |  |
| pRS-BCH2-F | AGAAGCAGCAACAGAAATACCAGCAG  CCATGAATtgtatatgagatagttgattgtatg |  |
| pRS-BCH2-R | CAGAAGGATCAAGAGGTCTAACGGTTC  TTGAACCTaagcttGTTAATTCAAATTAATTG |  |
| AcBCH1-F1 | CCCCTATTCGGAGATGTTTG | VIGS |
| AcBCH1-R1 | CTCCCACTTCTTCCAGTTCCTT |  |
| AcBCH2-F1 | CGGTTCACTTACCTTGTCGC |  |
| AcBCH2-R1 | ACTCTTCAAGCCCTCCCACT |  |
| pTRV1-F | TTACAGGTTATTTGGGCTGG |  |
| pTRV1-R | CCGGGTTCAATTCCTTATC |  |
| pTRV2-F | GCCATTAGCGACATCTAAAT |  |
| pTRV2-R | CTAAGTCCACTCGTCCGTAA |  |
| pTRV2-AcBCH1-F | CGGACGAGTGGACTTAGATTCTG |  |
| pTRV2-AcBCH1-R | GTGTGGTGGATCTGGTGTGCCG |  |
| pTRV2-AcBCH2-F | CGGACGAGTGGACTTAGATTCTG |  |
| pTRV2-AcBCH2-R | ACTTGTCTGTGTGGTGGAGCTGG |  |
| AcBCH1-F2 | GGTCTAGAATGGTTGCCGGAATTTCCGT | Overexpression construct |
| AcBCH1-R2 | ACGAGCTCTCATGATGAACTGTTGGATAGT |  |
| AcBCH2-F2 | GGTCTAGAATGGCGGCCGGAATTTCCGT |  |
| AcBCH2-R2 | ACGAGCTCTCATGAACCGTTGGATCGT |  |
| pBI-BCH1-F | CAGGAAACAGCTATGACCATGA |  |
| pBI-BCH1-R | TCAAGTAGGGTACTTTAGCAA |  |
| pBI-BCH2-F | CAGGAAACAGCTATGACCATGA |  |
| pBI-BCH2-R | GGGTACTTTAGCAATGGGCCCC |  |
| NPTⅡ-F | CGGATCTGGATCGTTTCG | Detection of resistance genes |
| NPTⅡ-R | CGCTTGGTCGGTCATTTC |  |
| SlActin-qPCR-F | CCTCAGCACATTCCAGCAG | qRT-PCR primers of carotenoid synthesis genes in tomato |
| SlActin-qPCR-R | CCACCAAACTTCTCCATCCC |  |
| SlPSY-qPCR-F | AGAGGTGGTGGAAAGCAA |  |
| SlPSY-qPCR-R | TCTCGGGAGTCATTAGCAT |  |
| SlPDS-qPCR-F | AGCAACGCTTTTTCCTGATG |  |
| SlPDS-qPCR-R | TCGGAGTTTTGACAACATGG |  |
| SlZDS-qPCR-F | AGTGGTTTCTGTCTAAAGGTGG |  |
| SlZDS-qPCR-R | ACCGAGCACTCATGTTATCAC |  |
| SlCRTISO-qPCR-F | GTTGGTGGAGTTGGCGAGAT |  |
| SlCRTISO-qPCR-R | TCCCACAGCTTTGCCATTG |  |
| SlLCYb-qPCR-F | GTCCACTTCCAGTATTACCTCAG |  |
| SlLCYb-qPCR-R | TGTCCTTGCCACCATATAACC |  |
| SlCYCB-qPCR-F | TGTTATTGAGGAAGAGAAATGTGTGAT |  |
| SlCYCB-qPCR-R | TCCCACCAATAGCCATAACATTTT |  |
| SlBCH-qPCR-F | CTGCTCATTCGCTTCATCAC |  |
| SlBCH-qPCR-R | CGTCCCTCCTACTTCTTCCA |  |
| SlECH-qPCR-F | CCCGTTTAGTGGAGGGCC |  |
| SlECH-qPCR-R | CGAGAGCAATTGTAGCTTCCAA |  |
| SlZEP-qPCR-F | ATGATAGACCGCCAACCTTTAGTT |  |
| SlZEP-qPCR-R | CCATGCATCCCCCTTGAC |  |
| SlCCD-qPCR-F | CCCCTTTCTTCAGGGCAATT |  |
| SlCCD-qPCR-R | TCCGGCAGATGGCCTATAAC |  |
| SlNCED-qPCR-F | CATGAACTTGAACACCCTTTGC |  |
| SlNCED-qPCR-R | CGTTTCGAACGTAAACGCCT |  |

**Supplemental Table S2. Pearson's correlation between carotenoid-associated genes transcript levels and carotenoid content in ‘Jinshi 1’ kiwifruit.** The * are statistically significant at P < 0.05, and ** are statistically significant at P < 0.01

|  | β-carotene | β-cryptoxanthin | Zeaxanthin | Lutein |
| --- | --- | --- | --- | --- |
| *PSY* | 0.815** | -0.688* | 0.934** | 0.886** |
| *PDS* | 0.653 | 0.571 | 0.451 | 0.662 |
| *ZDS* | -0.111 | 0.275 | 0.130 | 0.004 |
| *CRTISO* | 0.339 | -0.565 | 0.313 | 0.389 |
| *LCYb1* | -0.495 | 0.349 | -0.332 | -0.388 |
| *LCYb2* | -0.717* | 0.903** | -0.543 | -0.672* |
| ***BCH1*** | **-0.618** | **0.824**** | -0.424 | -0.562 |
| ***BCH2*** | **-0.688*** | **0.875**** | -0.504 | -0.639 |
| *LCYe* | 0.934** | -0.950** | 0.926** | 0.962** |
| *ECH* | -0.153 | 0.383 | 0.110 | -0.046 |
| *ZEP* | -0.969** | 0.873** | -0.910** | -0.944** |
| *VDE* | 0.477 | -0.663 | 0.525 | 0.556 |
| *CCD* | -0.604 | 0.807** | -0.400 | -0.540 |
| *NCED* | -0.744* | 0.843** | -0.550 | -0.674* |


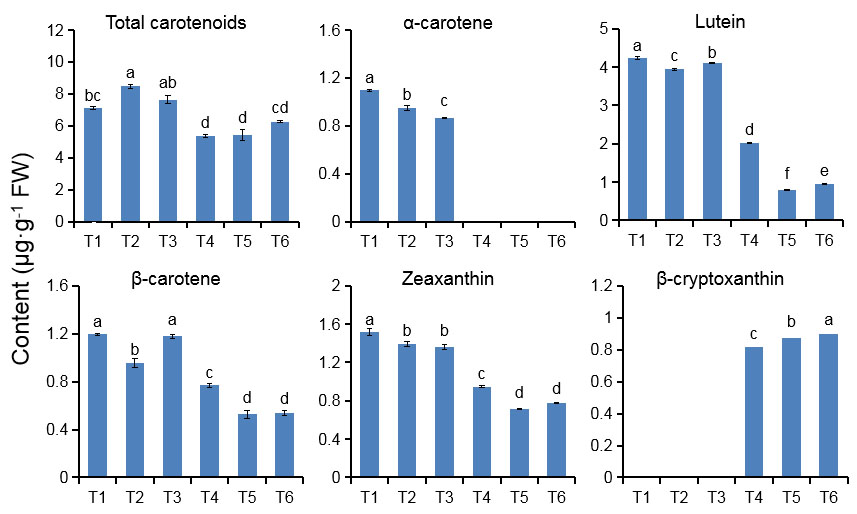


**Supplemental Figure S1. Carotenoid concentration in kiwifruit during fruit development.** T1-T6 represent 30, 55, 70, 95, 130 and 145 days after 75% flower drop.

**
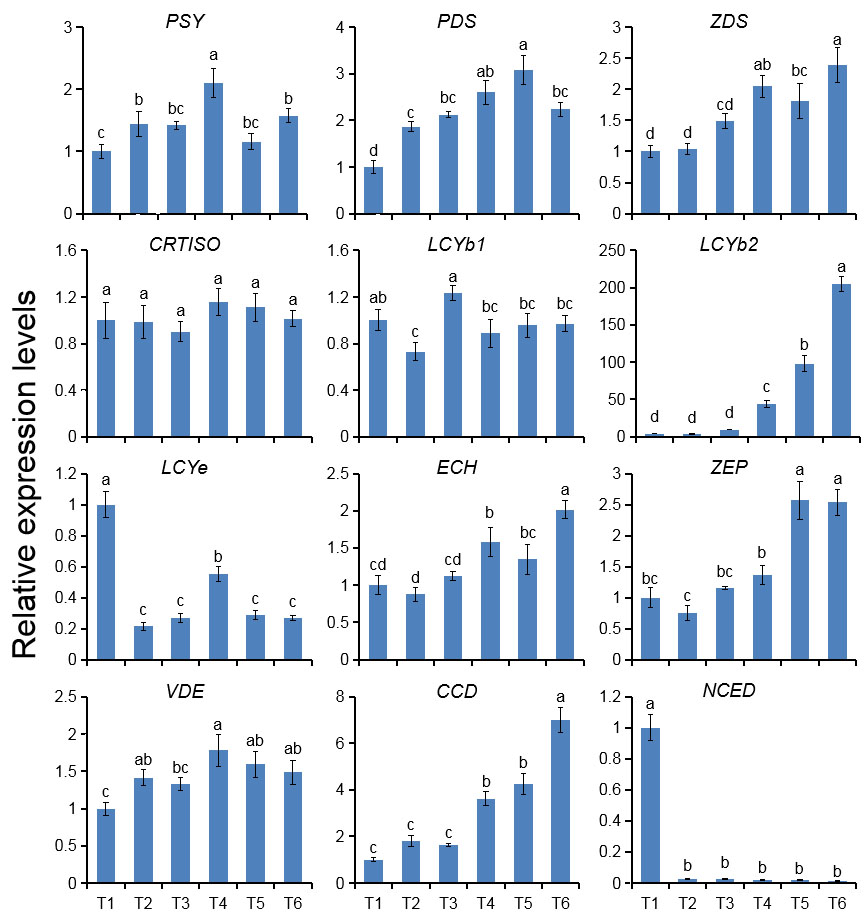
**

**Supplemental Figure S2. Relative expression levels of carotenoid-associated genes in kiwifruit during fruit development.** T1-T6 represent 30, 55, 70, 95, 130 and 145 days after 75% flower drop.
